# Facile Hydrophobication of Glutathione-Protected Gold Nanoclusters and Encapsulation into Poly(lactide-co-glycolide) Nanocarriers

**DOI:** 10.1038/s41598-019-47543-4

**Published:** 2019-07-31

**Authors:** Alaaldin M. Alkilany, Shrouq Alsotari, Mahmoud Y. Alkawareek, Samer R. Abulateefeh

**Affiliations:** 1 Department of Pharmaceutics & Pharmaceutical Technology, School of Pharmacy, The University of Jordan, Amman, 11942 Jordan; 2 Cell Therapy Center, The University of Jordan, Amman, 11942 Jordan

**Keywords:** Imaging techniques and agents, Materials science

## Abstract

We report a simple surface functionalization of glutathione-capped gold nanoclusters by hydrophobic ion pairing with alkylamine followed by a complete phase transfer to various organic solvents with maintained colloidal stability and photoluminescence properties. The described surface hydrophobication enables efficient encapsulation of gold nanoclusters into PLGA nanocarriers allowing their visualization inside cultured cells using confocal fluorescent microscopy. The simplicity and efficiency of the described protocols should extend the biomedical applications of these metallic nanoclusters as a fluorescent platform to label hydrophobic polymeric nanocarriers beyond conventional organic dyes.

## Introduction

Polymeric nanocarriers are employed in a wide range of biomedical and pharmaceutical applications due to their biocompatibility, biodegradability, and the availability of facile synthetic protocols to prepare them with excellent encapsulation efficiency and tunable release kinetics^1,2^. Of a particular interest, poly(lactide-co-glycolide) (PLGA) is a biocompatible and biodegradable polyester, which is used in several FDA-approved products in the form of microspheres and other pharmaceutical dosage forms^2,3^. Recently, more effort is devoted to develop PLGA-based nanoparticles to treat various pathologies such as cancer and inflammation as well as to provide superior vaccinations^4^. Understanding the nano-bio interaction of polymeric nanoparticles at the cellular and organ levels is a crucial fundamental and prerequisite to develop efficient and safe products. At this end, proper labeling of polymeric nanocarriers is required to enable their visualization via various imaging modalities. Fluorescence imaging is still considered the most used imaging tool to visualize cellular interaction of PLGA and other polymeric nanoparticle and has been recently extended to monitor their biodistribution *in vivo*^5^. Typically, organic florescent dyes are used due to their commercial availability, high quantum yield and the availability of documented protocol to encapsulate them into PLGA nanocarriers^6^. However, these florescent probes are susceptible to various drawbacks such as photo-bleaching and molecular leaching from the host, which may result in serious experimental artifacts^6–9^. To address these limitations, inorganic fluorescent nanoparticles such as inorganic quantum dots have been evaluated to label polymeric nanocarriers^9–11^. However, their low biocompatibility is limiting their biomedical applications due to the presence of toxic transition metal such as cadmium^12^. Alternatively, and more recently, gold-based fluorescent nanoclusters (AuNCs) attracted a significant interest as an ultrasmall inorganic nanoprobes (core size less than 2 nm) with stable photoluminescence properties^13–16^. Fundamentally, AuNCs are intriguing functional nanomaterials with a precise molecule-like structure (comprising a discrete number of gold atoms and protecting ligands) and considered as the missing link between molecules and nanoparticles^16^. A wide library of ligands was used to prepare these nanoclusters via aqueous and non-aqueous synthetic routes. AuNCs that are prepared using the aqueous route have typically a higher quantum yield and exhibit superior biocompatibility^16^. Among these AuNCs, glutathione and albumin protected-AuNCs are excellent candidates for a wide range of biomedical applications due to their biocompatibility, simple preparation in aqueous medium and excellent photoluminescence properties^17,18^. Glutathione-based gold NCs (GSH-AuNCs) are protected by a small peptide compared to the large albumin and thus exhibit small hydrodynamic diameter that enable renal excretion^19,20^. GSH-AuNCs can be synthesized in aqueous medium and exhibit relatively high quantum yield (15% relative to fluorescein^21^), large Stokes shift (>200 nm), chemical and photoluminescence stability^19,21^. The use of GSH-AuNCs as probes in conjunction of fluorescence and X-ray computed tomography (CT) imaging platforms has been reported *in vitro* and *in vivo*^17,19^. Moreover, and beyond their outstanding florescence-based applications, GSH-AuNCs have been evaluated recently as a photothermal^22^ and radio-sensitization^20^ therapeutic platforms for cancer treatment^20^. Motivated by these fascinating properties of GSH-AuNCs and in light of our interest to fabricate functional inorganic-organic nanohybrids^23^, we aim in this work to develop a facile protocol that allow an efficient encapsulation of GSH-AuNCs into PLGA nanocarriers and evaluate the feasibility of visualizing the labeled PLGA nanocarriers using confocal fluorescence microscopy.

## Results and Discussion

**Figure 1:**
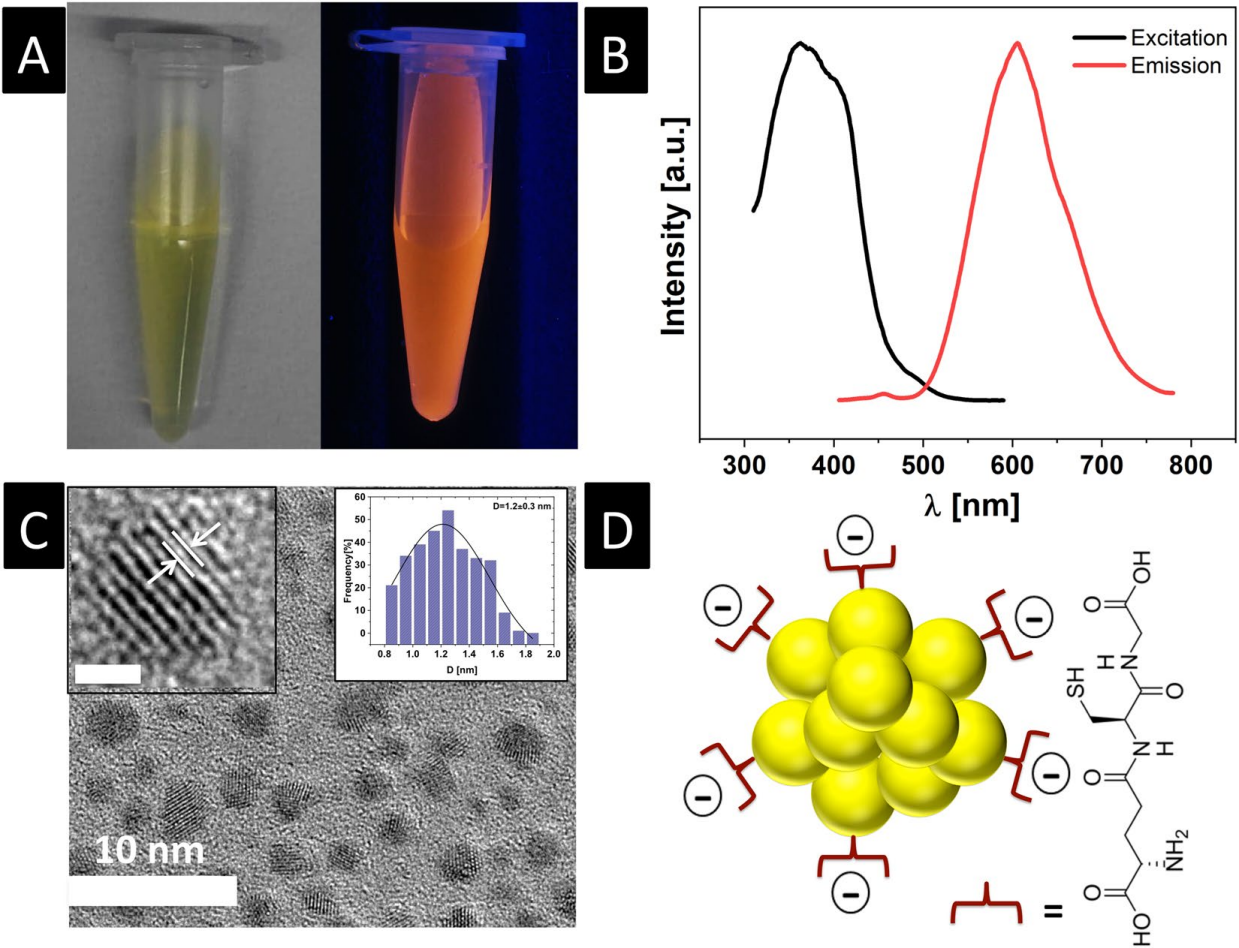
(**A**) Digital photograph of aqueous GSH-AuNCs suspension under daylight (left) and under long-wavelength UV lamp irradiation (365 nm) (right). (**B**) Excitation and emission spectra of GSH-AuNCs in water, as labeled. (**C**) TEM image of GSH-AuNCs (scale bar = 10 nm) with a left insert showing a high-resolution TEM image of the lattice planes separated by 0.24 nm (scale bar = 1 nm) and a right insert showing particle size histogram. (**D**) Sketch shows the gold core and glutathione shell of GSH-AuNCs as well as the chemical structure of the glutathione.

GHS-AuNCs was prepared using a reported protocol^21^ and resulted in a yellow suspension with a bright orange luminescence upon UV illumination (Fig. 1A). The prepared nanoclusters exhibited excitation and emission maximum around 365 nm and 605 nm, respectively (Fig. 1B), which matched well with the reported values^21^. High-resolution TEM imaging with imageJ analysis revealed an average core diameter of 1.2 ± 0.3 nm that is comparable to reported values^21^ (Fig. 1C) with a measured interfringe distance of 0.24 nm, which is consistent with lattice spacing of the face centered cubic (111) gold (Fig. S1, Supporting Information)^24^. Glutathione is a tri-peptide (L-g-glutamyl-L-cysteinyl-glycine) that acts as both reducing agent and capping agent, which assemble on the surface of AuNCs via a stable gold-thiol linkage (from cysteine) and imparts the nanoclusters with a net negative surface charge (zeta potential = −32 ± 4.2 mV, pH = 7.5) due to the presence of terminal carboxylic acid functional groups (sketch depicting the assembly of glutathione molecules on GSH-AuNCs and the chemical structure of glutathione peptide is shown in Fig. 1D).

PLGA is a hydrophobic polymer, which is more suited to encapsulate hydrophobic rather than hydrophilic agents via two general methods^4^: (1) Nanoprecipitation, in which PLGA and agents to be encapsulated are dissolved in a “good solvent” and then co-precipitate into nanoparticles upon addition into a non-solvent for both; (2) Emulsion-evaporation method in which PLGA and agents to be encapsulated are dissolved in a volatile water-immiscible organic solvent, which is emulsified into aqueous external phase followed by evaporation of the organic solvent to form PLGA nanoparticles. As prepared GSH-AuNCs are extremely hydrophilic and thus it is clear that surface hydrophobication of these nanoclusters is required for their efficient encapsulation into PLGA nanoparticles. Recently, we reported the necessity of hydrophobication of larger gold nanoparticles (15 nm) to ensure efficient encapsulation into PLGA nanocarriers^23^. Accordingly, we aimed first to develop a simple and efficient protocol to render these peptide-capped nanoclusters hydrophobic. It is worth to mention that available reports describing the encapsulation of GSH- or albumin-protected AuNCs are limited to hydrophilic, rather than hydrophobic, matrices such as nanogels^25^, aqueous core of microcapsules^26^, silica nanoparticles, protein nanoparticles^27,28^, polymer nanoparticles^29,30^ and metal-organic frameworks^31^. Accordingly, all mentioned examples imply the use of “as prepared” hydrophilic NCs with no surface modifications prior to their encapsulation.

**Figure 2:**
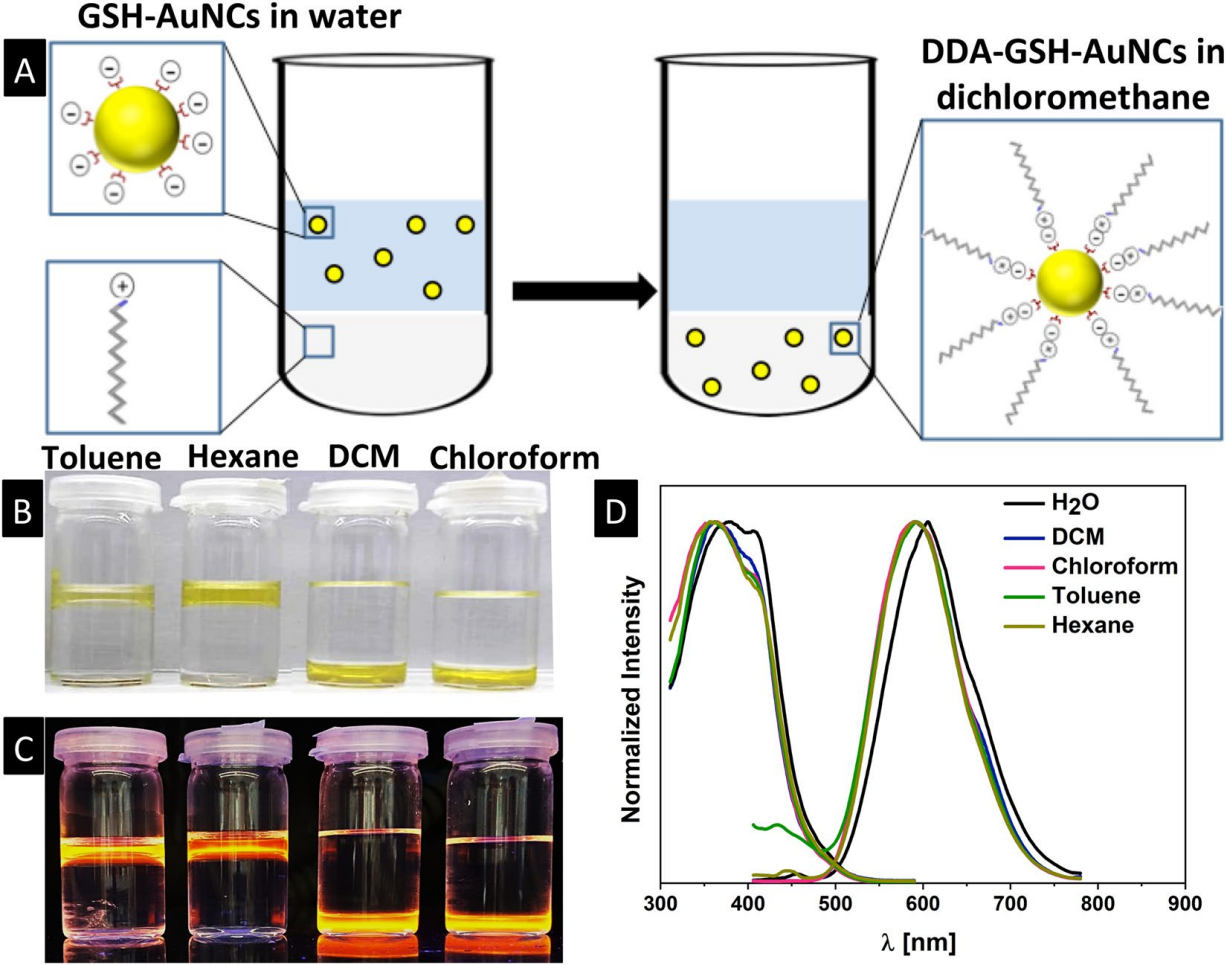
(**A**) Sketch shows hydrophobic ion pairing (between negatively charged carboxylic acid groups on GSH-AuNCs and positively charged amine groups on dodecylamine) and transfer of the resulting DDA-GSH-AuNCs from water to dichloromethane. (**B**) Digital photograph of vials contain biphasic system of water (clear layers) and immiscible organic solvents (yellowish layer, as labeled) contain transferred DDA-GSH-AuNCs. (**C**) Digital photograph of vials in B under long-wavelength UV lamp irradiation (365 nm). (**D**) Excitation and emission spectra of GSH-AuNCs in water and for DDA-GSH-AuNCs in various organic solvents as labeled.

Our initial attempts to hydrophobize the surface of GSH-AuNCs suggested a ligand exchange reaction to displace the glutathione molecules with a hydrophobic thiolated ligand (e.g. alkanethiols or thiolated-polymer). Unfortunately, these hydrophobic ligands are not soluble in water and hence require the addition of miscible organic co-solvents, which disrupts significantly the colloidal stability of GSH-AuNCs and results in severe and irreversible precipitation (unsuccessful attempts in this direction are discussed in Fig. S2, Supporting Information). Another challenge associated with ligand exchange on metallic nanoclusters is the possibility of altering the entire chemical structure and thus nanocluster’s size and photoluminescence properties^16,32–34^. With this in mind, we adapted an alternative approach to “hydrophobize” the surface of GSH-AuNCs that relies on the electrostatic interaction between hydrophobic cationic phase transfer agent (namely dodecylamine, DDA) and the negatively charged GSH-AuNCs. The hydrophobic ion pairing approach (HIP) is widely used to hydrophobize proteins and other biological molecules prior to formulate them into non-aqueous pharmaceutical preparations^35–37^. HIP alters the nanocluster’s hydrophobicity and suspendability in organic solvents without changing its molecular structure nor deteriorating its photoluminescence properties (In fact HIP may enhance the photoluminescence of NCs by shell rigidification mechanism)^38^. Moreover, HIP was proven as an excellent route to hydrophobize the surface of similar nanoclusters and to transfer them to organic solvents with maintained colloidal stability and photoluminescence properties^38–45^. Most available reports employ a bulky phase transfer agents (e.g. tetraoctylammonium bromide) to transfer GSH-AuNCs to toluene^38,44,45^, which is not the solvent of choice for PLGA nanoparticle preparation. A single report from the Bakr group describes the phase transfer of GSH-capped silver nanoclusters from water to dichloromethane (good solvent for PLGA preparation) using tetraphenylphosphonium bromide^32^. Motivated by these previous reports and the simplicity of the HIP approach, we evaluated the HIP of GSH-AuNCs with DDA (we avoided the use of quaternary ammonium phase transfer agents due to their toxicity). Our results indicate that DDA complexes with GSH-AuNCs and readily induces their complete transfer to chloroform or dichloromethane (typical solvents to prepare PLGA nanoparticles) as well as to other hydrophobic solvents (hexane and toluene) as shown in Fig. 2. In our system, HIP relies on the electrostatic pairing between anionic carboxylic acid groups of GSH-AuNCs and the positively charged amine groups in dodecylamine as sketched in Fig. 2A. The phase transfer of GSH-AuNCs by DDA to the organic solvents can be achieved equally by two methods: 1) addition of DDA in methanol to GSH-AuNCs in water to form a milky suspension followed by addition of the water-immiscible organic solvent to extract the DDA-GSH-AuNCs or 2) starting with a biphasic system of GSH-AuNCs in water and a water-immiscible organic solvent containing DDA followed by addition of methanol. In both methods, the addition of methanol was important to induce a complete phase transfer of DDA-GSH-AuNCs into the organic layer, in agreement with previous reports in which common solvents were used to facilitate phase transfer of nanoclusters and nanoparticles across biphasic liquid systems^32,46–49^. Both protocols were found equivalent and resulted in complete transfer of GSH-AuNCs to the organic layers without precipitation, aggregation or assembly of nanoclusters at the interface (Fig. 2B,C). The amount of DDA was found an important parameter as low levels fail to induce a complete phase transfer where at high levels the formation of undesired stable emulsions was observed with dependence on the used organic solvent (details are available in Fig. S3). It is worth to mention that the HIB of GSH-AuNCs with DDA and their phase transfer to organic solvents can be scaled up successfully (Fig. S3E). The maintained typical yellow color of GSH-AuNCs (Fig. 2B) and main features in their optical absorption spectra (Fig. S4) confirms the retention of the gold core in the evaluated organic solvents upon the phase transfer reaction. Moreover, the transferred DDA-GSH-AuNCs maintain their fluorescence properties in all evaluated organic solvents as shown in Fig. 2C,D. Comparing the photoluminescence profile of GSH-AuNCs before and after phase transfer revealed a slight blue shift in their emission maxima in all evaluated solvents (Fig. 2D and Table S1), which may be attributed to rigidification of the GSH surface ligands due to the HIP with DDA. Similar blue shift was observed previously when GSH-AuNCs was bound to bulky surfactant (tetraoctylammonium bromide) to rigidify the Au(I)-GSH shell^38^. Moreover, the solvent effect (solvatochromic shift) is expected to contribute to the observed blue sift in the emission spectra of transferred DAA-GSH-AuNCs in organic solvents.

**Figure 3:**
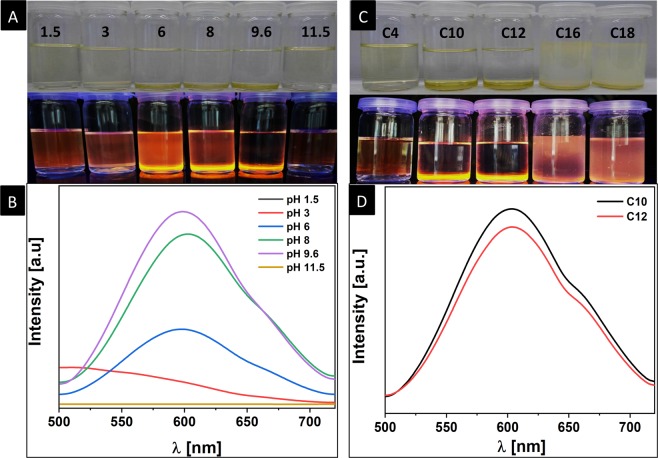
Effect of pH and the alkane chain length on the phase transfer of GSH-AuNCs into dichloromethane using dodecylamine. (**A**) Digital photograph of vials contain biphasic system of water and dichloromethane under daylight (upper panel) and long-wavelength UV lamp irradiation (365 nm) (lower panel) upon phase transfer at various pH of the aqueous phase (methanol/DDA was added to the aqueous GSH-AuNCs and then the pH was adjusted to the labeled value on the graph). (**B**) Emission spectra of DDA-GSH-AuNCs in dichloromethane (vials in **A**) upon phase transfer at various pH. (**C**) Digital photograph of vials contain biphasic system of water and dichloromethane under daylight (upper panel) and long-wavelength UV lamp irradiation (365 nm) (lower panel) upon phase transfer using either butylamine (C4), decylamine (C10), dodecylamine (C12), hexadecylamine (C16) or octadecylamine (C18). (**D**) Emission spectra of transferred GSH-AuNCs using decyamine (C10) or dodecylamine (C12) in dichloromethane.

To confirm the electrostatic interaction between DDA and GSH-AuNCs, the phase transfer at optimum DDA level was evaluated as function of pH. Interestingly, we found that efficient phase transfer from water to dichloromethane only occurs in the pH range of 6.0–9.6, at which the amine groups in DDA are protonated (positively charged) and the carboxylic acid groups in GSH are deprotonated (negatively charged) supporting the electrostatic nature of the HIP in DDA-GSH-AuNCs (Fig. 3A,B). Furthermore, no phase transfer was observed at extremely basic (11.5) or acidic (1.5 and 3) pH values at which either the amine groups in DDA or the carboxylic acid groups in GSH molecules are not charged, respectively (Fig. 3A,B).

Furthermore, since the alkane chain of DDA should be the origin of the hydrophobic character of alkylamine-GSH AuNCs, we studied the hydrophobication and phase transfer of GSH-AuNCs using alkylamines with various chain length (C4, C10, C12, C16 and C18) from water to dichloromethane. At equal molar concentrations (based on the optimized amount for DDA), we found that both C10 and C12 (DDA) are equivalent and both induced a facile phase transfer while C4 did not. Phase transfer with C16 and C18 resulted in the formation of highly stable and viscous emulsion, which hampered the transfer of AuNCs (Fig. 3C,D).

**Figure 4:**
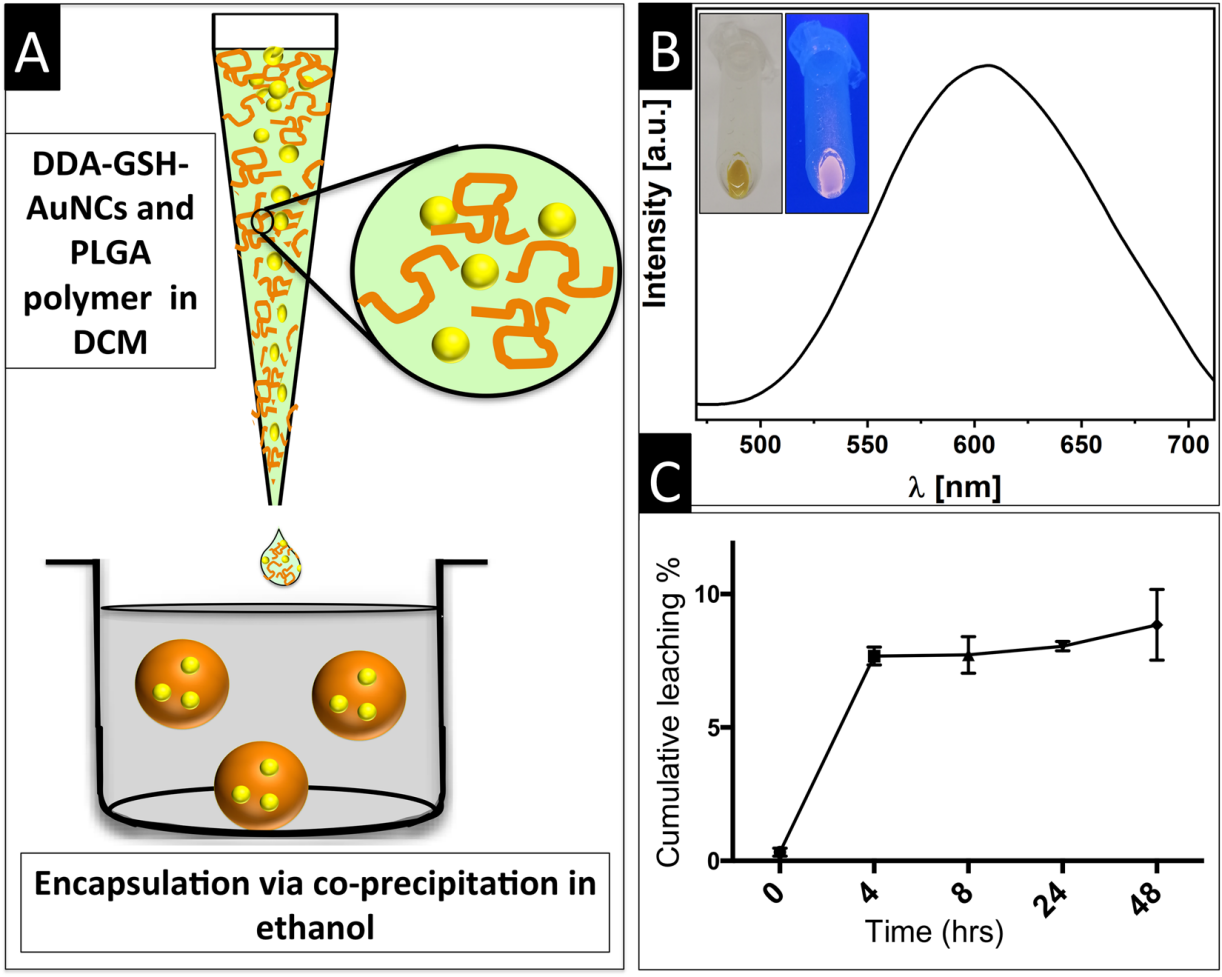
(**A**) Sketch shows encapsulation of DDA-GSH-AuNCs into PLGA nanoparticles using the nanoprecipitation method. (**B**) Florescence spectra of PLGA nanoparticles encapsulating DDA-GSH-AuNCs (Insert: digital photograph of pellets of PLGA nanoparticles encapsulating DDA-GSH-AuNCs under daylight (left) or long-wavelength UV lamp irradiation (365 nm) (right)). (**C**) Leaching of encapsulated DDA-GSH-AuNCs from PLGA nanocarriers as function of time. Total leaching was calculated as percent to initial load of DDA-GSH-AuNCs in to PLGA nanoparticles. Values represent average and standard deviation of three independent experiments.

Successful hydrophobication of GSH-AuNCs and their excellent colloidal stability in organic solvents encouraged us to evaluate their encapsulation into PLGA nanoparticles using a modified nanoprecipitation method and the emulsion-evaporation method, as detailed earlier in the manuscript. For the nanoprecipitation method, PLGA polymer was dissolved in a suspension of DDA-GSH-AuNCs in dichloromethane (DCM) (after the phase transfer) and the resulting mixture was added drop wise into ethanol (non-solvent for PLGA and non-suspending medium for DDA-GSH-AuNCs) resulting in the formation of PLGA nanoparticles loaded with DDA-GSH-AuNCs (cartoon for the encapsulation process is sketched in Fig. 4A). Selection of DCM/ethanol system for the nanoprecipitation method was based on an evaluation of the suspendablity of DDA-GSH-AuNCs after phase transfer in various solvents that include good and poor solvents for PLGA as detailed in Fig. S5. For the emulsion-evaporation method, PLGA polymer was dissolved in a dispersion of DDA- GSH-AuNCs in dichloromethane, which was then emulsified into aqueous external phase. Evaporation of the organic solvent leads to the formation of PLGA nanoparticles encapsulating the hydrophobic DDA-GSH-AuNCs. Our preliminary experimentation suggested that both methods are equivalent and thus we employed the nanoprecipitation method due to its superior simplicity. In a typical encapsulation experiment, PLGA nanoparticles exhibited a hydrodynamic diameter of 170 ± 30 nm (z-average, PDI = 0.162) as measured using dynamic light scattering with anionic effective surface charge (−20.5 ± 5.3 mV) due to the terminal carboxylate groups on the PLGA polymers. Moreover, suspensions of PLGA nanoparticles encapsulating DDA-GSH-AuNCs emit orange/red upon illumination (Fig. 4B). Encapsulation efficiency was measured using ICP-MS and revealed excellent encapsulation efficiency of DDA-GSH-AuNCs into PLGA matrix (93.09 ± 1.71%), owing to the hydrophobic character of the nanoclusters that prevents its partition into the external polar phase (ethanol) and rather get encapsulated in the hydrophobic PLGA matrix. Tendency of encapsulated AuNCs to leach out from the PLGA hosts was evaluated as a function of incubation time using ICP-MS. Our data reveled excellent encapsulation stability of DDA-AuNCs in PLGA with very minimal leaching as shown in Fig. 4C (total leaching was 8.85 ± 1.32% after 48 hrs). The leached amount would be attributed to the poor association/adsorption of a small fraction of the AuNCs with/at the surface of PLGA in resemblance to the known “burst release” of encapsulated drugs in PLGA nanohosts. For comparison with leaching of molecular dye, similar PLGA nanoparticles were loaded with Nile red, a popular dye used for labeling PLGA and other polymers^50^, and found to leach 32.2 ± 6.7% of initial load after only 6 hrs. The retarded leaching of nanoclusters can be attributed to their large size in comparison with molecular dyes and it is a clear advantage for imaging purposes as dye leaching decreases the fluorescence brightness of NPs and increases the background signal resulting in unfavorable imaging inefficiency/artifacts^50^.

**Figure 5:**
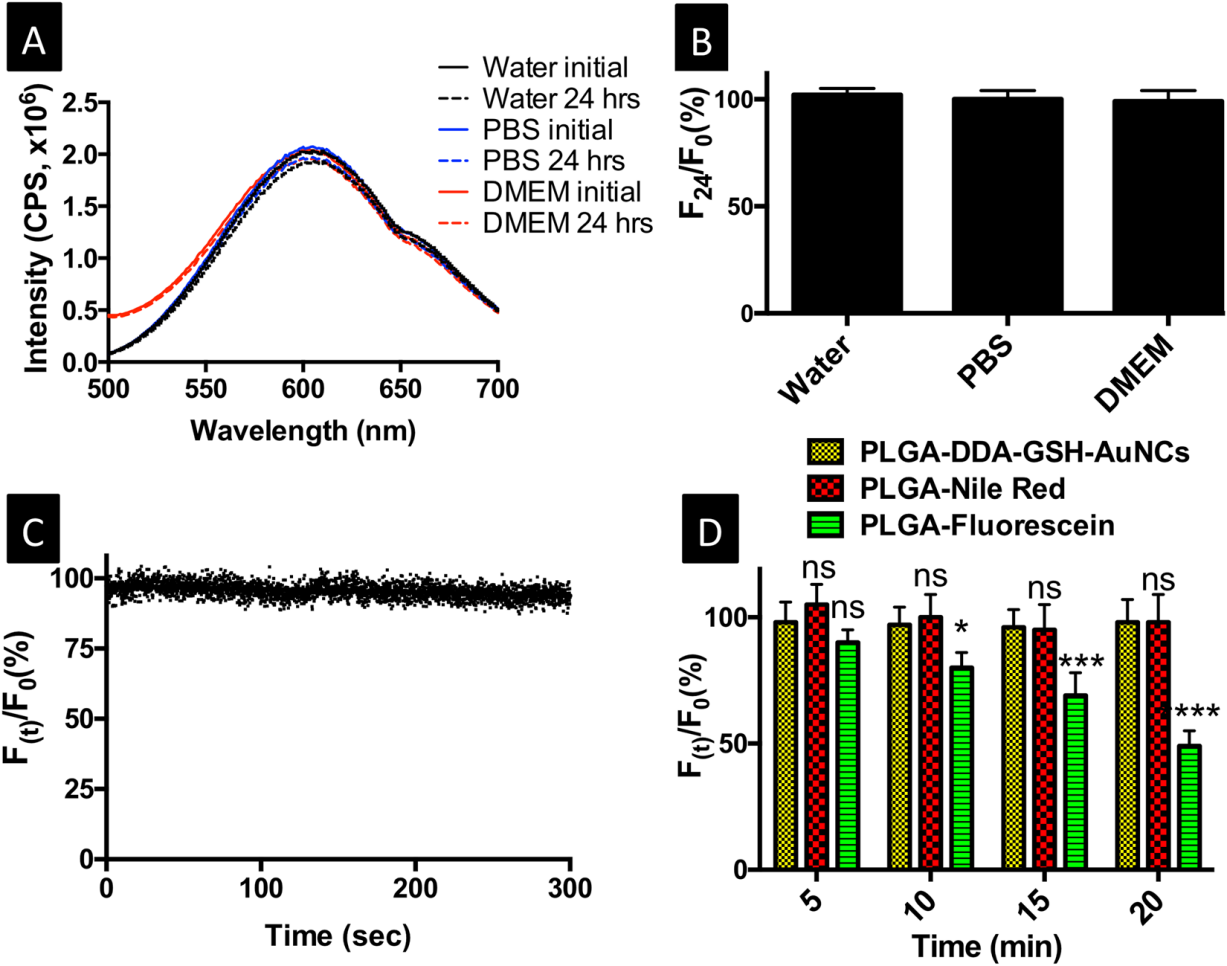
Photostability of PLGA nanoparticles encapsulating DDA-GSH-AuNCs. (**A**) Photoluminescence spectra and (**B**) relative photoluminescence intensity calculated as the ration between intensity after 24 hours of incubation (F_24_) to the initial intensity (F_0_) in water, PBS or DMEM as labeled. **C**) Photostability of PLGA nanoparticles encapsulating DDA-GSH-AuNCs in DMEM upon kinetic scan (λ_ex_ = 365 nm, λ_em_ = 605 nm, integration time of 0.1 s, total scan time of 300 S, 150 W xenon lamp). **D**) Photostability of PLGA nanoparticles encapsulating DDA-GSH-AuNCs (yellow bars), Nile Red (red bars) or fluorescein (green bars) upon irradiation with (364 nm, 80 mW, 248 mW/cm^−2^), (568 nm, 30 mW, 93 mW/cm^−2^) or (488 nm, 30 mW, 93 mW/cm^−2^), respectively. In (**C**,**D**): F_0_ and F_(t)_ are the initial photoluminescence intensity before irradiation and at each time point as labeled, respectively. Data in (**B**) and (D) are mean ± SD, n = 3 for each group. Statistical analysis in (**D**) was performed by 2-way ANOVA with the Bonferroni post-hoc test. In (**D**), significant difference of PLGA-Nile red or PLGA-Fluorescein in reference to PLGA-DDA-GSH-AuNCs is calculated using (alpha of 0.05) and indicated by: *p < 0.5; ***p < 0.001; ****p < 0.0001; ns: not significant (p > 0.05).

Photostability and biocompatibility of fluorescent nanoprobes are prerequisites for their biomedical applications. In this regard, we evaluated possible fluorescence quenching upon mixing PLGA nanoparticles encapsulating DDA-GSH-AuNCs with cell growth media, which typically contain various ions, small molecules and proteins. Figure 5A,B confirm a stable photoluminescence of PLGA nanoparticles encapsulating DDA-GSH-AuNCs in PBS or DMEM (Dulbecco’s Modified Eagle Medium) contains 10% FBS (fetal bovine serum) for 24 hours with comparable photoluminescence stability in water as a control. Furthermore, PLGA nanoparticles encapsulating DDA-GSH-AuNCs in DMEM resist photobleaching upon recording a continuous scan (kinetic mode, total scans of 3000, λ_ex_ = 365 nm, λ_em_ = 605 nm, 150 W xenon lamp) as evident from a constant photoluminescence signal during scanning as shown in Fig. 5C. Moreover, the resistance of PLGA nanoparticles encapsulating DDA-GSH-AuNCs to photobleach upon continuous laser irradiation was evaluated in comparison with conventional fluorescent dyes: Nile red and fluorescein (relatively stable and labile dyes to photobleaching, respectively)^51^. Upon 20 minutes of laser irradiation, PLGA nanoparticles encapsulating either DDA-GSH-AuNCs or Nile red retained their initial photoluminescence intensity with no significant loss, where the labile fluorescein lost half of its initial photoluminescence intensity (Fig. 5D). The resistance of encapsulated AuNCs to photobleach in our study agrees with an increasing body of literature supporting the outstanding photostability of gold nanoclusters^16,52–54^. For examples, Sun *et*. *al*. confirmed the photostability of GSH-AuNCs upon irradiation with 450 W xenon lamp as ~80% of initial intensity were preserved even after 180 minutes of continuous irradiation^52^. Similarly, Yu *et*. *al* reported a maintained photoluminescence properties (more than 85% of original emission) of chitosan protected AuNCs after continuous UV irradiation for 15 minutes^53^.

**Figure 6:**
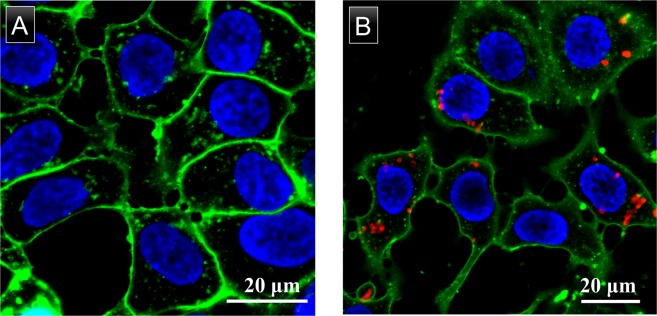
Confocal fluorescence microscopy images of MCF-7 cells treated with (**A**) plain PLGA nanoparticles (control) and (**B**) PLGA nanoparticles labeled with DDA-GSH-AuNCs. Nuclei were stained with Hoechst 33342 (blue color) and cell membranes were stained with CellMask orange (green color). Red pixels are emitted fluorescence of encapsulated DDA-GSH-AuNCs in the dosed PLGA nanocarriers. Scale bars: 20 μm.

*In vitro* toxicity of PLGA nanoparticles encapsulating DDA-GSH-AuNCs were evaluated in comparison with empty PLGA nanoparticles (PLGA as a biocompatible and FDA approved material)^3^. The encapsulation of DDA-GSH-AuNCs into PLGA nanoparticles does not alter the well documented biocompatibility of PLGA as evident from similar cellular viability of MCF-7 cells upon exposure to empty PLGA nanoparticles or PLGA nanoparticles encapsulating DDA-GSH-AuNCs in a wide range of nanoparticle concentration (4–500 μg/mL) as shown in Fig. S6. Our results can be explained by the well documented biocompatibility of GSH-AuNCs *in vitro*^29,55^ and *in vivo*^19^. As a proof of concept, PLGA nanoparticles encapsulated with DDA-GSH-AuNCs were dosed to cultured cells (MCF-7) to evaluate the ability to visualize their uptake using confocal fluorescence microscopy. Figure 6 shows a clear uptake of labeled PLGA nanocarriers with AuNCs with bright red photoluminescence at loading capacity of 0.79 weight % (gold/polymer, determined using ICP-MS analysis). In conclusion, we have developed a facile protocol to hydrophobize GSH-AuNCs and to efficiently encapsulate them into PLGA nanocarriers with maintained photoluminescence properties and minimal leaching. Collectively, our results confirm the outstanding photostability, minimal leaching and biocompatibility of PLGA nanoparticles encapsulated with DDA-GSH-AuNCs and suggest the use of gold nanoclusters as an alternative fluorescent platform to conventional dyes in labeling polymeric nanocarriers for biomedical imaging.

## Methods

### Synthesis of GSH-AuNCs

Synthesis of GSH-AuNCs was adapted from published protocol^21^. Briefly, aqueous solution of HAuCl_4_ (1.0 mL, 20 mM) was mixed with aqueous solution of reduced glutathione (0.3 mL, 100 mM) with gentle stirring. Then, solution was brought to total volume of 10 mL by adding 8.7 mL of milliQ water (solution color will turn to dark orange immediately and will turn gradually to colorless with time). The mixture was incubated at 70 °C for 24 with gentle mixing. Final solution color is yellow.

### Phase transfer of GSH-AuNCs with DDA to organic solvents

As prepared GSH-AuNCs (1.0 mL) was diluted with 2.0 mL of milliQ water. Then methanol (3.0 mL) containing dodecylamine (50 mg) was added with mixing (white yellowish suspension was formed). Organic solvent (1.0 mL of either hexane, toluene, chloroform or DCM) was added to extract the DDA-GSH-AuNCs to the organic phase.

### Encapsulation of DDA-GSH-AuNCs into PLGA nanoparticles using the nanoprecipitation method

First GSH-AuNCs was transfer to dichloromethane as described above. Dichloromethane layer can be used “as is” to dissolve PLGA polymer or can be dried using rotary evaporator and fresh dichloromethane is added to suspend the DAA-GSH-AuNCs and dissolve PLGA. In both ways, PLGA (25 mg) was dissolved in dichloromethane containing DDA-GSH-AuNCs (1.0 mL), and then the dispersion was added drop wise into stirring ethanol (20.0 mL). The formation of hazy yellowish suspension indicated the formation of PLGA nanoparticles encapsulating gold nanoclusters. The suspension was dried using rotary evaporator and the resulting film was washed with ethanol to remove excess dodecylamine. The dried film was then hydrated with milliQ water to suspend PLGA nanoparticles. Hydrodynamic diameter and zeta potential values of prepared PLGA nanoparticles were measured using Zetasizer Nano-ZS (Malvern instruments, UK).

### Encapsulation of DDA-GSH-AuNCs into PLGA nanoparticles using the emulsion-evaporation method

PLGA (30 mg) was dissolved in chloroform containing DDA-GSH-AuNCs (1.5 mL), and then emulsified into aqueous PVA solution (2.5% w/w, 5.0 mL) using sonication for 60 s (ultra-probe sonicator at 50 W, UP200Ht, Hielscher Ultrasonics, Teltow, Germany). The resulting emulsion was left stirring overnight to evaporate the organic solvent leading to the formation of PLGA nanoparticles loaded with AuNCs. The resulting nanoparticles were purified by centrifugation at 19354 × g for 10 minutes (Centurion Scientific Ltd., UK). The supernatant was discarded while the sediment nanoparticles were washed firstly with water to remove excess PVA and then with ethanol to remove excess dodecylamine. Each washing step was followed by centrifugation as mentioned above. The supernatant was discarded while the sediment nanoparticles were re-suspended in milliQ water.

### Encapsulation of Nile red or fluorescein to PLGA nanoparticles

To PLGA solution in acetone (1.0 mL, 25 mg/mL) either Nile red or PLGA-fluorescein was dissolved (0.2% w/w) and then the resulting solution was added drop wise into stirring water (5.0 mL).

### Calculation of encapsulation efficiency

After a typical encapsulation experiment, PLGA pellets were collected by centrifugation at 12396 g for 20 minutes using a micro centrifuge (Eppendorf 5418, Hamburg, Germany). ICP-MS analysis was used to determine the gold amount in the initial DDA-GSH-AuNCs suspension (*m*_*initial*_) and PLGA nanoparticle pellet (*m*_*p*_) as well as in the supernatant. Encapsulation efficiency percentage (EE%) was calculated according to the equation below:

$$EE \% =\frac{{m}_{p}\,}{{m}_{initial}\,}\times 100 \% $$

The mass balance of AuNCs amount analyzed for initial loading, pellets and supernatants samples was >95%.

### Leaching experiment

Immediately, after a typical encapsulation experiment, aqueous suspension of PLGA nanoparticles loaded with AuNCs was left stirring at 400 rpm and 25 °C. At specific time points (0, 4, 8, 24 and 48 hours), 1.0 mL of nanoparticle suspension was centrifuged at 19354 × g for 10 minutes (Centurion Scientific Ltd., UK). Collected pellets (containing PLGA nanoparticles) and supernatant (containing leached AuNCs as they do not sediment at this centrifugation conditions) were analyzed using ICP-MS. At each time point, the amount of leached AuNCs was divided by the amount of the initially loaded cluster and expressed as % cumulative leaching. The mass balance of AuNCs amount analyzed for initial loading, pellets and supernatants samples at any time point was >95%.

### ICP-MS analysis

The concentration of gold in samples was measured with ICP-MS according to previous report^23^. For the sample preparation, 150 µL of freshly prepared aqua regia (HCl:HNO_3_ = 3:1) was added to 50 µL of the sample, and the mixture were kept in an auto-shaker for overnight at room temperature. Afterwards, 1.8 mL of milliQ water was added (final solution volume = 2 mL and the dilution factor is 40). Elemental concentrations of gold where then determined using mass spectrometer (Perkin-Elmer, ELAN DRC-e).

### Viability assay

In 96-Well microplate, MCF-7 cells were incubated with various concentrations of PLGA nanoparticles (4–500 μg/mL) either empty (control) or encapsulating DDA-GSH-AuNCs (gold content of 0.79% w/w of PLGA) in Dulbecco’s Modified Eagle’s Medium (DMEM) with 10% FBS for 24 hours. Then, 20 µL of MTT solutions was added to each well followed by an incubation period of 3 hours, then the media were removed and 200 µl of DMSO was added to dissolve the crystal and the absorbance was recorded at 570 nm (BioTek, USA) and cell viability was calculated as a percentage compared to cells treated with DMEM alone.

### Photostability evaluation

To test the photostability in buffer and cell culture media, PLGA nanoparticles encapsulating DDA-GSH-AuNCs in water (2 mg/mL) was mixed with water (control), PBS or DMEM that contains 10% FBS (final concentration of PLGA nanoparticles is 0.5 mg/mL and AuNCs 4 μg/mL as gold). Photoluminescence spectra were collected initially and after 24 hours of incubation at 37 °C and relative photoluminescence intensity was calculated as the ration between intensity after 24 hours of incubation to the initial intensity in water, PBS or DMEM was calculated.

To test the Photostability upon laser irradiation, 200 µL of PLGA nanoparticles encapsulating DDA-GSH-AuNCs, Nile red or fluorescein were placed in UV 96-Well microplate and irradiated with the following lasers: (364 nm, 80 mW, 248 mW/cm^−2^), (568 nm, 30 mW, 93 mW/cm^−2^) and (488 nm, 30 mW, 93 mW/cm^−2^), respectively using confocal laser scanning microscope (LSM 510 META, Carl Zeiss MicroImaging GmbH, Göttingen, Germany) for total duration of 20 minutes. At each time point (five minutes intervals), photoluminescence spectra was recorded using a FluoroMax 4 (Horiba, Jobin Yvon GmbH, Unterhaching, Germany) spectrophotometer working with a continuous 450 W xenon lamp at λ_ex_/λ_em_ of: 365/605, 552/636 and 495/519 nm for PLGA encapsulating DDA-GSH-AuNCs, Nile red or fluorescein, respectively. Relative photoluminescence intensity was then calculated from the ration between intensity at each time point to the initial intensity before irradiation.

### Fluorescence imaging

MCF-7 cells were incubated with either plain PLGA nanoparticles (0.1 mg/mL) or PLGA nanoparticles labeled with AuNCs (0.1 mg/mL PLGA, 0.8 μg/mL AuNCs as gold) for 24 h in 10% FBS containing cell culture medium before imaging with confocal laser scanning microscope. Nuclei were stained with Hoechst 33342 and cell membranes were stained with CellMask orange. Scale bar, 20 μm.

## Supplementary information

Revised ESI
