# Supplementary material for: Facile Hydrophobication of Glutathione-Protected Gold Nanoclusters and Encapsulation into Poly(lactide-co-glycolide) Nanocarriers
Source: Sci Rep. 2019 Jul 31;9:11098. doi: 10.1038/s41598-019-47543-4 (PMC6668383; doi:10.1038/s41598-019-47543-4)
Supplement: Supplementary file 1 — Revised ESI [file 41598_2019_47543_MOESM1_ESM.docx]

Supporting Information

**Facile Hydrophobication of Glutathione-Protected Gold Nanoclusters and Encapsulation into Poly(lactide-co-glycolide) Nanocarriers**

*Alaaldin M. Alkilany^1*^, Shorouq T. Alsoutari^2^, Mahmoud Y. Alkawareek^1^, Samer R. Abulateefeh^1^*

^1^Department of Pharmaceutics & Pharmaceutical Technology, School of Pharmacy, The University of Jordan, Amman 11942, Jordan

^2^Cell Therapy Center, The University of Jordan, Amman 11942, Jordan

* Corresponding author: [a.alkilany@ju.edu.jo](mailto:a.alkilany@ju.edu.jo)

**Chemicals:**

Chloroauric acid (HAuCl_4_·3H_2_O, 99.9%), PLGA (Resomer® RG 504 H; Lactide to glycolide ratio is 50:50; MW ca. 38-54 kDa (used for the preparation of PLGA nanoparticles), poly(L-lactide), thiol terminated, dodecylamine, 1-dodecanethiol, polyvinyl alcohol (PVA, Mw. 31-50 kDa), Nile Red, L-Glutathione (reduced) and DMEM (without phenol red, #D1145) were obtained from Sigma-Aldrich and used as received. PLGA-fluorescein was purchased from nanosoft polymers (MW ca. 10 KDa, Lactide to glycolide ratio is 50:50). All solutions/dispersions were prepared with milliQ water. Glassware was cleaned with aqua regia and rinsed thoroughly with milliQ water.


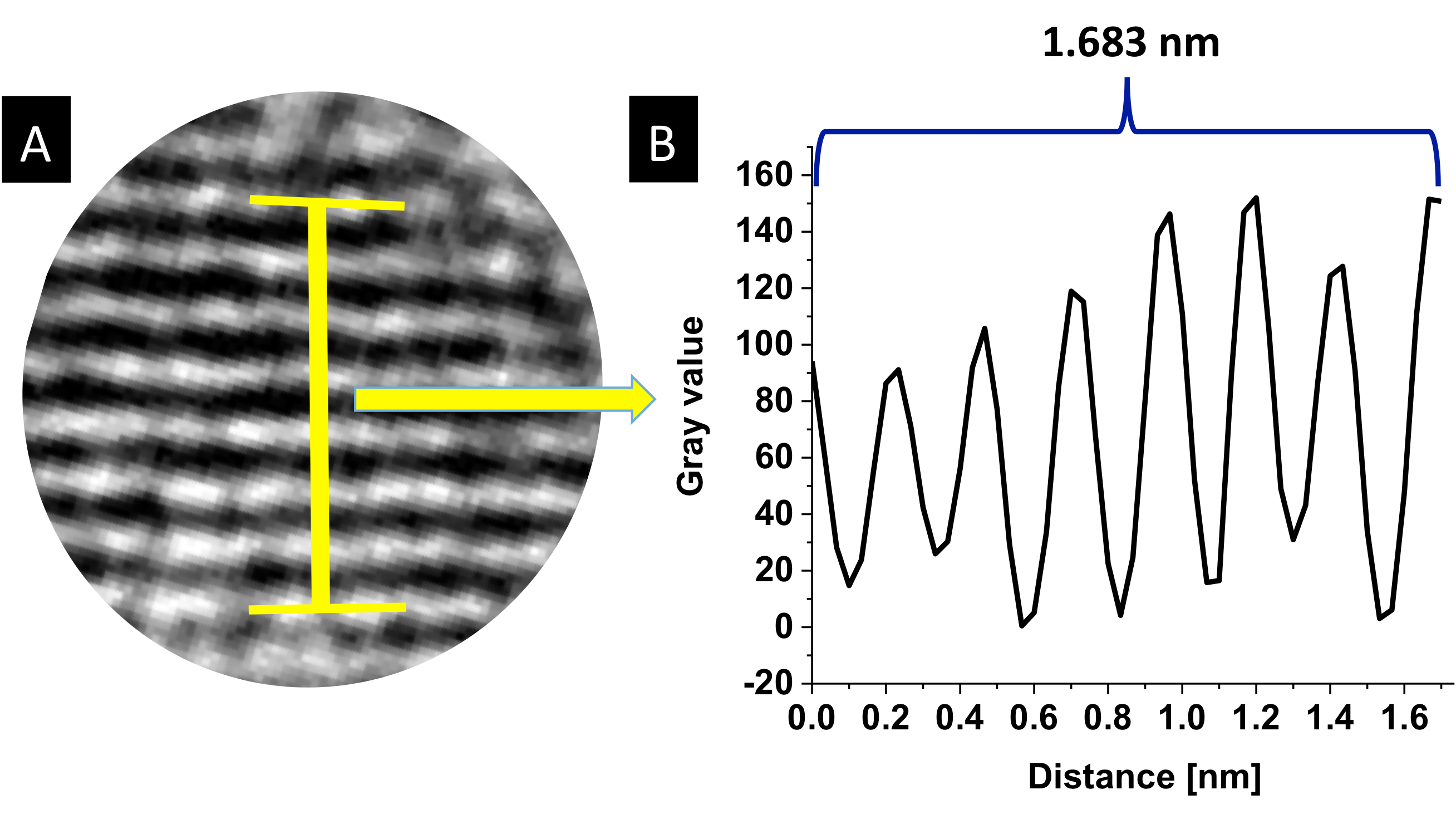


**Figure S1.** A) HRTEM image of prepared GSH-AuNCs (selected region of Figure 1C in main manuscript) with a yellow line that is drawn perpendicular to few atom planes for analysis. B) Image J analysis suggest the presence of seven atom planes (number of cycles of gray value in (B) with a corresponding total interplanar distance of 1.683 nm. Single interplanar spacing can be calculated by dividing the total interplanar distance by the number of atom planes, which result of an average value of 0.24 nm that agree with interplanar distance value for Au 111 pane.


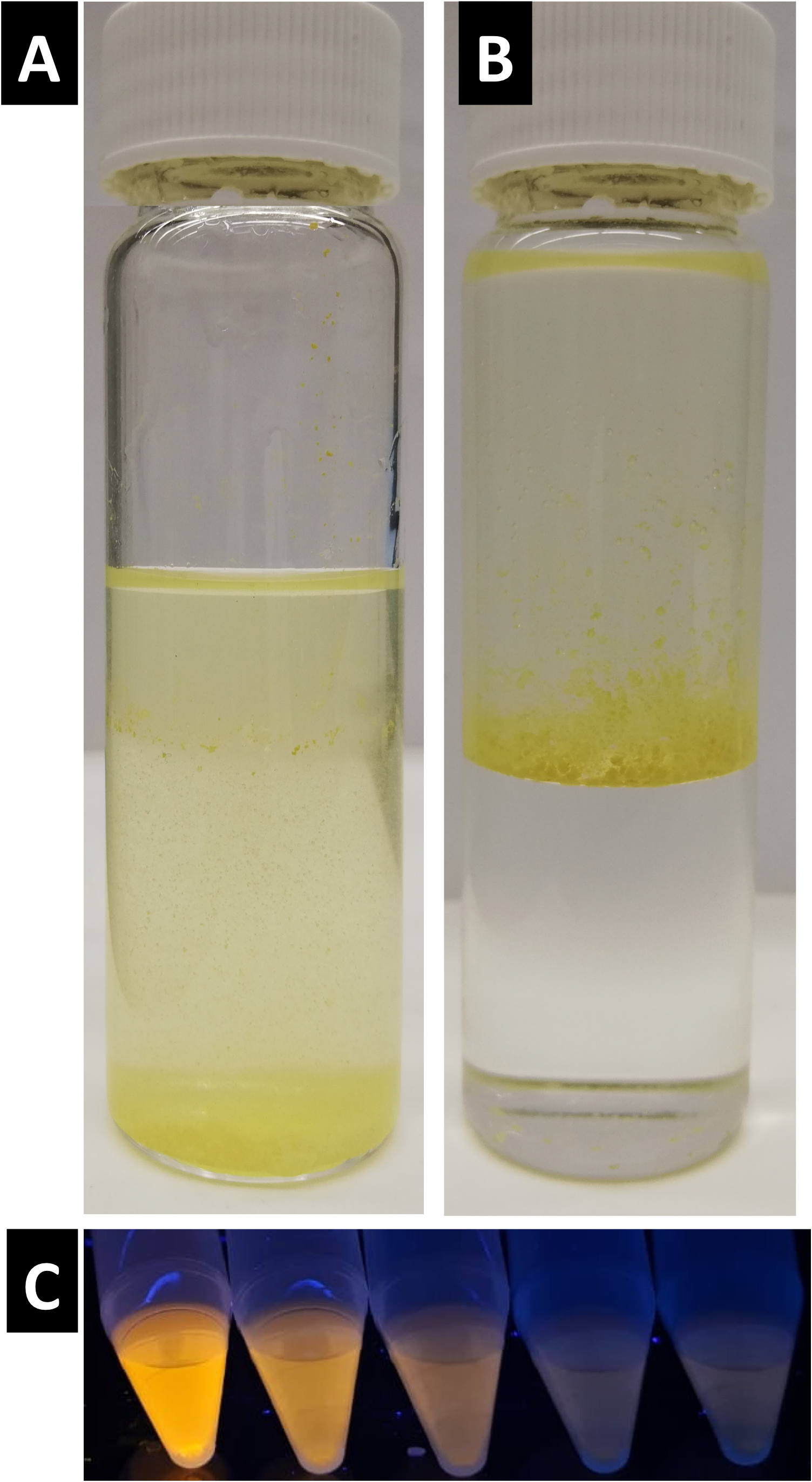


**Figure S2.**  Representative unsuccessful attempts to hydrophobize GSH-AuNCs using the ligand exchange route. A) An attempt to exchange GSH with hydrophobic PLA-SH (hydrophobic polymer) was conducted in a single phase of water-DMF system (1:5 v/v), which is necessary to ensure the solubility of PLA-SH. GSH-AuNCs aggregated in this system as evident from the yellow precipitate at the bottom of the vial. Similar results were observed when acetone, acetonitrile or DMSO were used as a solvent instead of DMF. B) Another attempt to exchange GSH with hydrophobic alkanethiols (dodecanethiol, DDT). Biphasic system of water-dichloromethane mixture (1:1 v/v) with DDT as a phase transfer agent and acetone as a common solvent. Various amounts of DDT (0-100 μL/mL in the dichloromethane) and types of common solvents (methanol, ethanol, acetone, DMSO, DMF and THF) evaluated and failed to induce a successful phase transfer. In fact, aggregation of GSH-AuNCs and their assembly at the water-dichloromethane interface is observed. C) Ligand exchange-induced photobelaching of GSH-AuNCs. Digital photograph of DDA-GSH-AuNCs in dichloromethane under long-wavelength UV lamp irradiation (365 nm) after typical phase transfer with DDA followed by addition of DDT in various amounts to exchange GSH (incubation time of 12 hours, 60 ^o^C). Concentration of DDT from left to right: 0 (control), 2, 4, 8, 16 μL neat DDT/50 mg DDA-GSH-AuNCs.


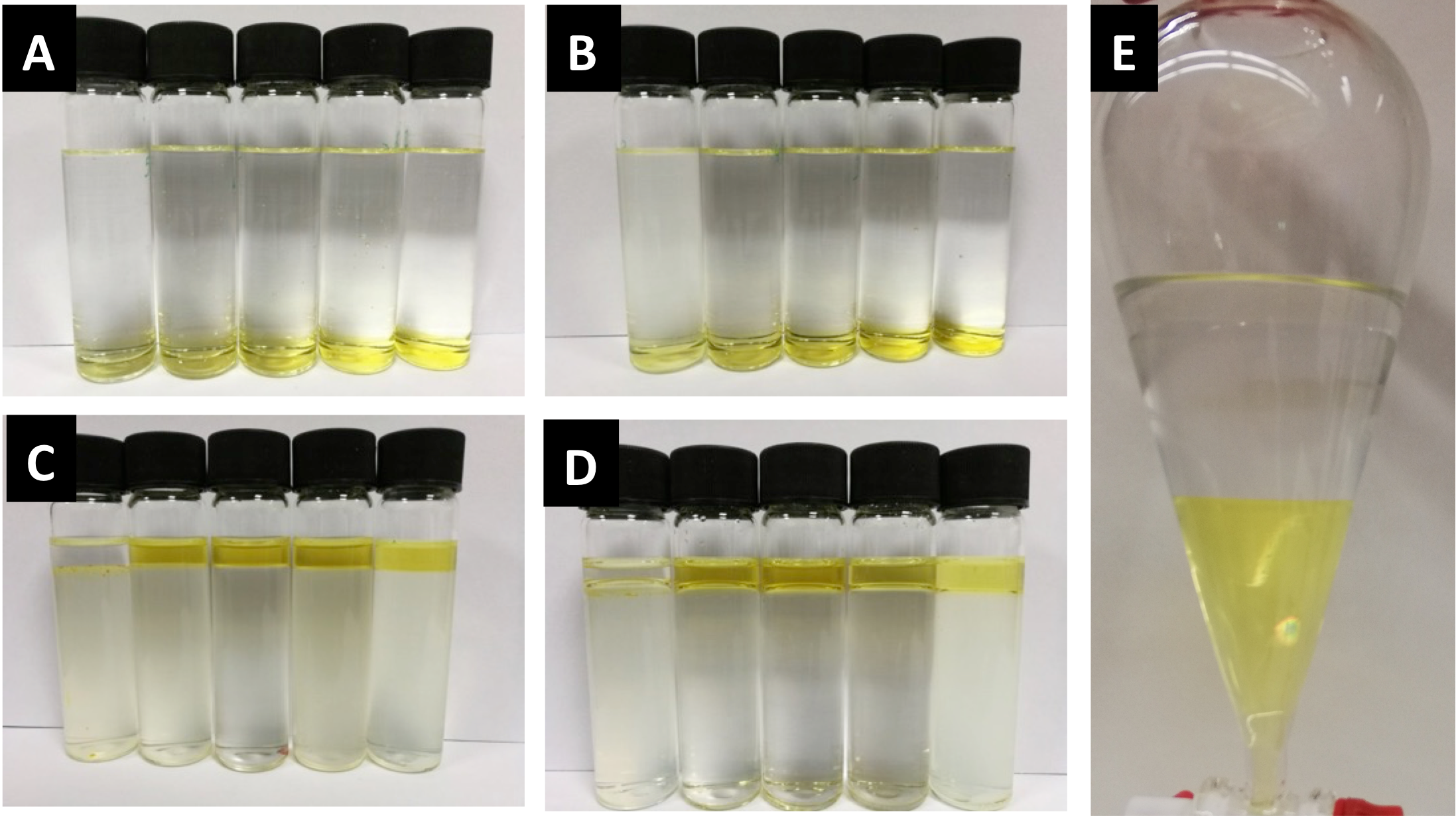


**Figure S3**. Digital photograph of vials contain biphasic system of water (clear layers) and immiscible organic solvents (yellowish layer) contain transferred DDA-GSH-AuNCs. Organic solvents are: A) chloroform, B) dichloromethane, C) hexane and D) toluene. Concentration of DDA from left to right in all photos is: 5, 10, 25,50 and 100 mg per mL of GSH-AuNC (as prepared). Note that: 1) 5 mg/mL was not enough to transfer all GSH-AuNCs to hexane or toluene but this level of DDA induced a complete phase transfer when chloroform and dichloromethane was used; 2) at high DDA level (100 mg), stable emulsions were formed in the case of hexane and toluene but not in chloroform or dichloromethane, which retarded a complete phase transfer of GSH-AuNCs. E) Scale up of the phase transfer. Digital photograph of a separatory funnel contains a biphasic system of water (upper clear layer) and dichloromethane (lower yellow layer) upon phase transfer of GSH-AuNCs (50 mL) from water to dichloromethane using dodecylamine.


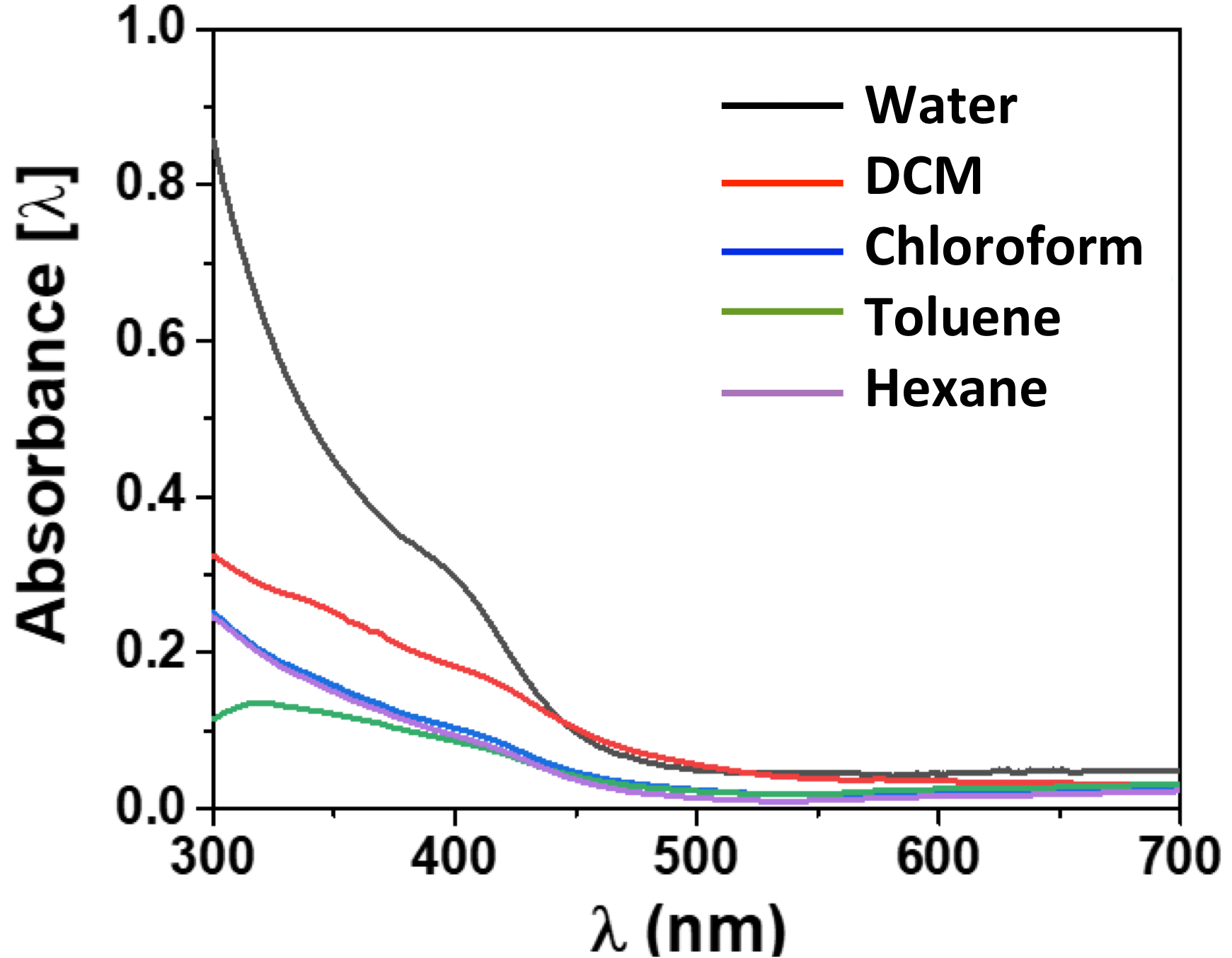


**Figure S4.** Absorbance spectra of DDA-GSH-AuNCs in various organic solvents upon phase transfer with DDA as labeled.

**Table S1.** Excitation and emission maximum values (λ_ex_ and λ_em_. respectively) of GSH-AuNCs in water and for DDA-GSH-AuNCs in various organic solvents as labeled.

| **Solvent** | **λ_ex_ (nm)** | **λ_em_ (nm)** |
| --- | --- | --- |
| **Water** | 378 | 606 |
| **DCM** | 363 | 593 |
| **Chloroform** | 365 | 590 |
| **Toluene** | 362 | 593 |
| **Hexane** | 356 | 590 |

**
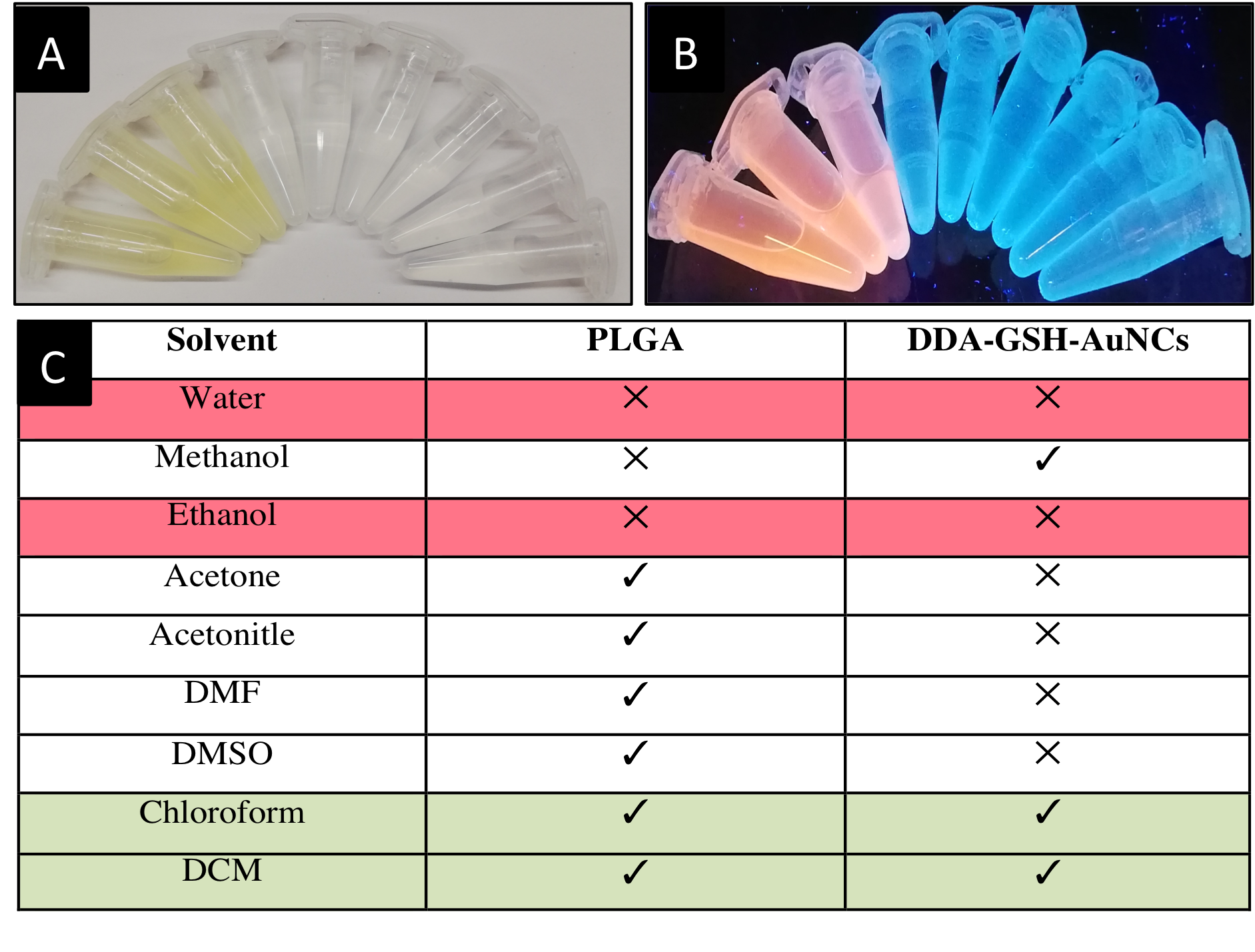
**

**Figure S5.** Suspendability of DDA-GSH-AuNCs in various solvents. After typical phase transfer, DDA-GSH-AuNCs in dichloromethane was placed into centrifuged tubes and the solvent was allowed to evaporate in chemical hood at room temperature in open air. The resulting powder received various solvents, mixed vigorously and incubated overnight. The dispersion was then centrifuged to separate fraction not dispersed (5 minutes, 2000 g). Supernatant were placed in clean tubes and imaged using digital camera under day light (A) and long-wavelength UV lamp irradiation (365 nm) (B). Solvents in A and B from left to right: dichloromethane, chloroform, methanol, water, ethanol, acetone, acetonitrile, DMF, DMSO. Owing to the hydrophobicity of DDA-GSH-AuNCs, they exhibit excellent suspendability in hydrophobic solvents (dichloromethane and chloroform). Surprisingly, DDA-GSH-AuNCs also found to exhibit suspendability in methanol (explanation is not clear but maybe due to the high solubility of DDA in methanol which may induce partial dissolution of DDA from the GSH-AuNCs). The suspendability behavior of DDA-GSH-AuNCs in various solvents parallel to the solubility of PLGA in same solvents are summarized in C. Green rows indicate “good solvents” for PLGA and good dispersing medium for DDA-GSH-AuNCs. Red rows indicate “poor solvents” for PLGA and poor dispersing medium for DDA-GSH-AuNCs, Based on these observations, DCM and ethanol was selected to perform all encapsulation experiments (water was excluded due to immiscibility with both DCM and chloroform) via our modified nanoprecipitation process.


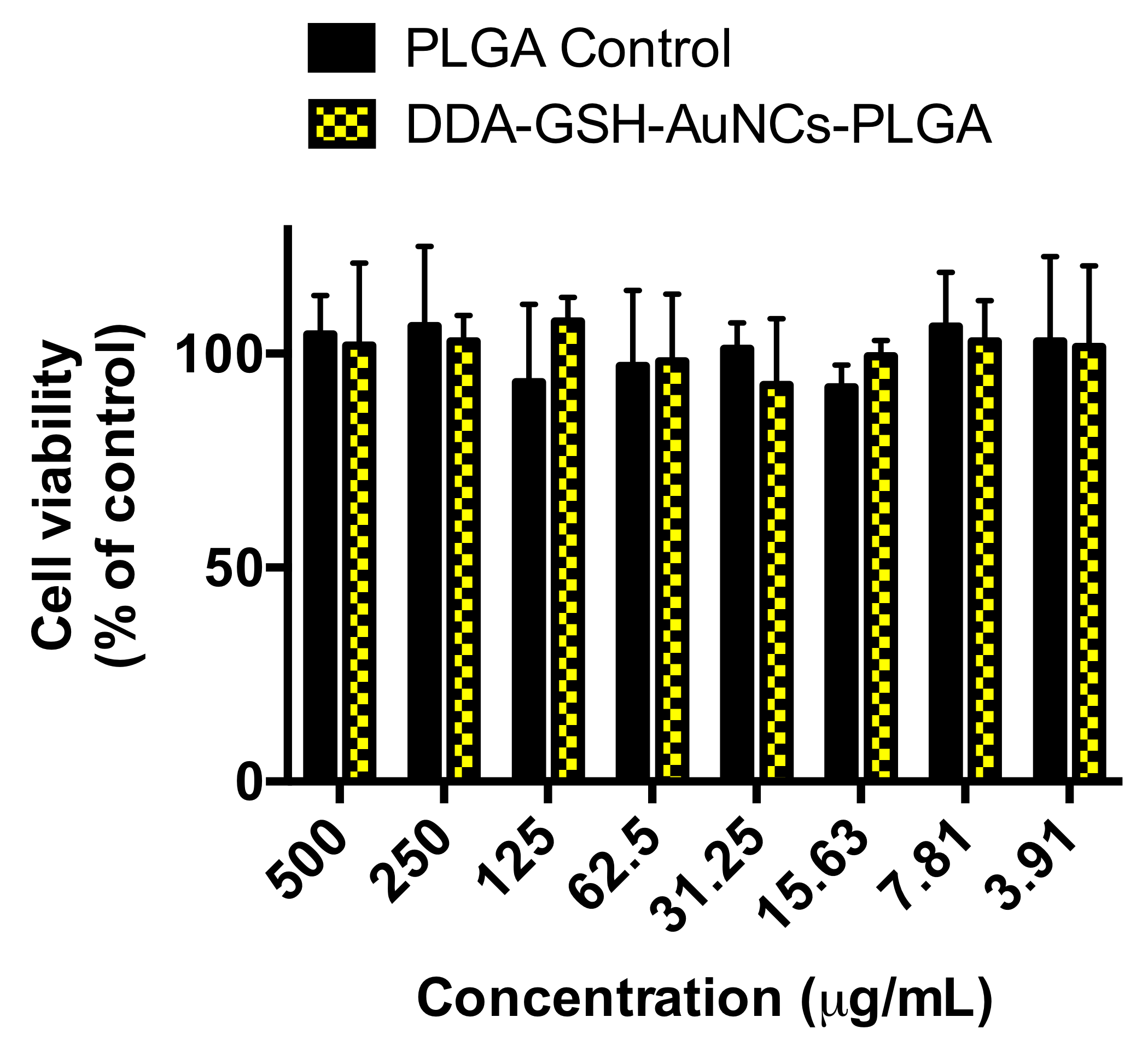


**Figure S6.** Viability of MCF-7 cells upon exposure to empty PLGA nanoparticles (black bars, control) or PLGA nanoparticles encapsulating DDA-GSH-AuNCs (yellow bars) at various nanoparticle concentrations.
